# Both Systemic and Intra-articular Immunization with Citrullinated Peptides Are Needed to Induce Arthritis in the Macaque

**DOI:** 10.3389/fimmu.2017.01816

**Published:** 2017-12-20

**Authors:** Samuel Bitoun, Pierre Roques, Thibaut Larcher, Gaétane Nocturne, Che Serguera, Pascale Chrétien, Guy Serre, Roger Le Grand, Xavier Mariette

**Affiliations:** ^1^Rheumatology Department, Université Paris-Sud, AP-HP, Hôpitaux Universitaires Paris-Sud, INSERM U1184, Le Kremlin Bicêtre, France; ^2^Immunology of Viral Infections and Autoimmune Diseases, IDMIT Infrastructure CEA, Université Paris-Sud, INSERM U1184, Fontenay-Aux-Roses, France; ^3^INRA UMR703 Veterinary School of Nantes, Nantes, France; ^4^Modélisation des Biothérapies MIRCen, CEA/INSERM US27, Fontenay-Aux-Roses, France; ^5^Immunology Department AP-HP, Hôpitaux Universitaires Paris-Sud, Le Kremlin Bicêtre, France; ^6^“Epithelial Differentiation and Rheumatoid Autoimmunity” Unit, INSERM U1056, Université de Toulouse, Toulouse, France

**Keywords:** rheumatoid arthritis, ACPA, neutrophils, citrulline, shared epitope, macaque

## Abstract

**Objectives:**

Anti-citrullinated peptides antibodies (ACPAs) have high specificity for the diagnosis of rheumatoid arthritis (RA), but their role in the pathophysiology is not fully established. The main genetic risk factor for RA, the shared epitope in major histocompatibility complex class II, is associated with ACPAs. Among certain non-human primates, 8% carry the shared epitope called H6 haplotype, and being similar to humans, are ideal candidates to study the role of ACPAs in RA. The goal of this study was to develop a macaque model of RA based on immunization against citrullinated peptides to generate an ACPA-mediated model of arthritis.

**Methods:**

Cynomolgus macaques were immunized with four citrullinated peptides from vimentin, fibrinogen, and aggrecan, known to induce T-cell response in RA patients, and received an intra-articular (IA) boost with the same four citrullinated peptides pooled.

**Results:**

In the macaque, the T-cell response was specific to citrullinated peptides. Antibodies generated in response to immunization were cross-reactive between the citrulline and arginine peptides. The presence of the H6 haplotype did not affect the magnitude of the immune response. Since no clinical response was observed, macaques received an IA boost with the same four peptides pooled and incomplete Freund’s adjuvant, which led to a prolonged neutrophil-rich mono-arthritis, preferentially in H6-positive animals. Conversely, animals boosted with incomplete Freund’s adjuvant alone presented only transient mono-arthritis.

**Conclusion:**

This two-hit model of prolonged mono-arthritis mimics what could happen in RA. Despite the limited number of joints with disease in the macaque model, the model appears unique to study the events occurring during the preclinical phase of RA, from immunization against citrullinated peptides to the clinical appearance of disease.

## Introduction

Rheumatoid arthritis (RA) is a debilitating disease that affects the joints and causes severe handicap. Posttranslational modification of arginine to citrulline residues occurs in a wide range of proteins during various inflammatory processes and might trigger an immune response against citrullinated epitopes generating autoantibodies (1). In RA, antibodies against citrullinated peptides (ACPAs) are frequent (75% of patients) and specific to the disease (up to 98% diagnosis specificity). This very high specificity for RA has led to the inclusion of ACPAs in the latest disease diagnostic criteria from the American College of Rheumatology/European League Against Rheumatism (ACR/EULAR 2010) (2).

The current paradox is that despite the high diagnostic value of ACPAs, their implication in the pathophysiology of RA is not fully established. For instance, in transgenic mice expressing the human major histocompatibility complex (MHC) class II shared epitope, arthritides have been induced with citrullinated peptides (3), but these results have been poorly reproduced and remain controversial (4). In humans, the shared epitope confers increased risk of RA and was also thought to increase ACPA level. The link has been suggested to rely on the increased affinity of some citrullinated peptides for the human leukocyte antigen (HLA) molecule as compared with the native unmodified peptides (5).

In a species of macaque, *Macaca fascicularis*, 8% naturally carry a version of the shared epitope. Previous macaque models of RA relied only on collagen immunization (6), which usually led to rapid severe joint destruction mimicking acute arthritis (7), which is far from the mechanism of RA pathogenesis in humans.

The purpose of this study was to set up an experimental macaque model of RA closer to the human disease than the currently available mouse models. Macaques carrying the shared epitope were immunized with different citrullinated peptides for presentation to T cells by the MHC molecule expressed in these animals. This model led to robust anti-citrullinated peptide T-cell and B-cell responses. A second-hit intra-articular (IA) injection of citrullinated peptides induced chronic articular inflammation only in previously immunized animals.

## Materials and Methods

### Study Design

Ten animals were used in this study. We immunized four H6 animals (carrying the shared epitope) with citrullinated peptides (*n* = 2) or native arginine peptides (*n* = 2) and two non-H6 animals were immunized with citrullinated peptides. Four control animals were used: two without systemic immunization and two with intradermal immunization with recombinant human myelin oligodendrocyte glycoprotein (rhMOG). A summary of the design is in Table S1 in Supplementary Material.

### Animals

Adult captive-bred 3- to 5-year-old female cynomolgus macaques (*M. fascicularis*) were used. The study was approved by the regional animal care and ethics committee (Comité Régional d’Ethique sur l’Expérimentation Animale Île de France Sud, Fontenay-Aux-Roses, France; decision #A13_026). The CEA Institute was approved as compliant with ETS123 recommendations for animal breeding (European Union Directive 2010/63/EU, September 22, 2010) and with Standards for Human Care and Use of Laboratory Animals (Animal Welfare Assurance, OLAW no. #A5826-01). The study was also approved by the French department of education and research (MENESR; study no. 02769.01) as defined in French law “décret 2013-118 from 2013 Feb 1st.” At the end of each study, sedated animals were euthanized by intravenous injection of a lethal dose of pentobarbital.

### Genotyping

Cynomolgus macaques (*M. fascicularis*) from Mauritius have a well-characterized genetic background and limited MHC diversity because of their isolation. Microsatellite analysis revealed six common haplotypes (H1–6) of MHC class II (8). The H6 genotype in the DRB1 region shares the same amino acid sequence as the 70–74 sequence of the human shared epitope DRB1 04-04 (QRRAA) (Table S2 in Supplementary Material). H6 animals were identified by a DNA microsatellite technique as described in Ref. (9). Presence of the shared epitope was confirmed after whole-blood RNA extraction with two specifically designed RT-PCR primers and probes followed by sequencing (Table S3 in Supplementary Material). Thus, the amino acid sequence on the macaque *Mafa* class II DRB alleles in positions 11 and 70–74 could be precisely identified.

### Peptide Selection

Several proteins identified as targets of ACPAs include fibrinogen, vimentin, aggrecan, and enolase. Certain peptides from these proteins can induce a citrulline-specific T-cell response in patients with RA who carry the shared epitope (5, 10–12). We chose four peptides that are presented by DRB1 04-04 (5). These peptides originating from vimentin 59-71 (Vim59), vimentin 66-78 (Vim66) fibrinogen alpha chain 79-91 (Fg79), and aggrecan 89-103 (Agg89) (Table S4 in Supplementary Material) were synthesized in native or citrullinated form, the arginyl substituted by citrullyl residues (Genscript, Piscataway, NJ, USA).

### Immunization

Monkeys were immunized intradermally, each peptide injected individually, at 500 μg/peptide. Peptides were adjuvated with CpG and Montanide^®^ (kindly provided by Seppic, Puteaux, France) and injected in the back of the animals every 2 weeks for a total of four injections.

### Immune Response

Peripheral blood mononuclear cells (PBMCs) were collected, and the T-cell response was assessed by comparing treatment with the pool of four citrullinated peptides used for immunization of animals to that of native peptides. T-cell response against citrullinated peptides and against arginine peptides was assessed using a macaque anti-interferon-gamma (IFNγ) Enzyme Linked Immunospot (ELISPOT) kit (Mabtech, Nacka Strand, Sweden). In total, 200 000 cells were exposed to a pool of 2 µg/mL of each peptide for 18 h. Positive controls were phorbol myristate acetate and ionomycin. Negative controls (cells with medium only) were subtracted from the sample for each animal. Positive spots were defined as 40 wide with 20 intensity using an AID EliSpot reader (Autoimmun Diagnostika, Strassberg, Germany). B-cell response against citrullinated peptides and arginine peptides was assessed using an in-house-designed ELISA (13). Briefly, peptides were passively coated overnight. After washing, blocking was performed for 1 h at 4°C with 2% bovine serum albumin (BSA). After washing, serum diluted in 2% BSA was incubated for 1 h at 4°C. Goat anti-monkey IgG (BioRad, Watford, UK) was then added to wells and revealed using 3,3′,5,5′;-tetramethylbenzidine (Lifetech, Villebon Sur Yvette, France). Results are displayed as ratio of optical densities to the preimmunization state for each animal. Ratios >2 were considered positive. Cytokine analysis was performed on plasma collected in heparin lithium tubes using a non-human primate 23-cytokine multiplex assay (Millipore, Guyancourt, France, Table S5 in Supplementary Material).

### IA Injection

Animals were injected in the right knee with the citrullinated peptides Vim59, Vim66, Fg79, and Agg89 with or without incomplete Freund’s adjuvant (IFA) (Sigma-Aldrich, Saint-Quentin-Fallavier, France). Control animals were injected with IFA alone. Other control animals previously immunized with rhMOG (1–125) were injected intra-articularly with rhMOG + IFA. See experimental procedure in Method S6 in Supplementary Material.

### Measurement of Clinical and Inflammatory Response

Clinical inflammation was scored from 0 to 4. Systemic inflammation was measured by plasma C-reactive protein (CRP) level. Blood counting was performed at each blood sampling using an HMX Hematology analyzer (Beckman Coulter, Villepinte, France).

### Histology

Tissue samples were fixed in 4% formalin, embedded in paraffin wax or 2-hydroxyethyl(glycol)methacrylate, then transversally cut into 5-μm thick sections. Sections were stained by a routine hematoxylin–eosin–safranin staining method. All samples were evaluated by a skilled pathologist in a double-blind manner, and all lesions were systematically recorded. Serial additional sections of the knee joint were stained with safranin O/fast green for better observation of osteochondral structures (Histalim, Toulouse, France).

### Statistics

When applicable, data were compared by non-parametric tests, and continuous values were compared by Mann–Whitney tests with GraphPad Prism 7. *P* < 0.05 was considered statistically significant.

## Results

### Citrullinated Peptide Immunization Leads to a Citrulline-Specific Non-H6-Restricted T-Cell Response

**Figure 1:**
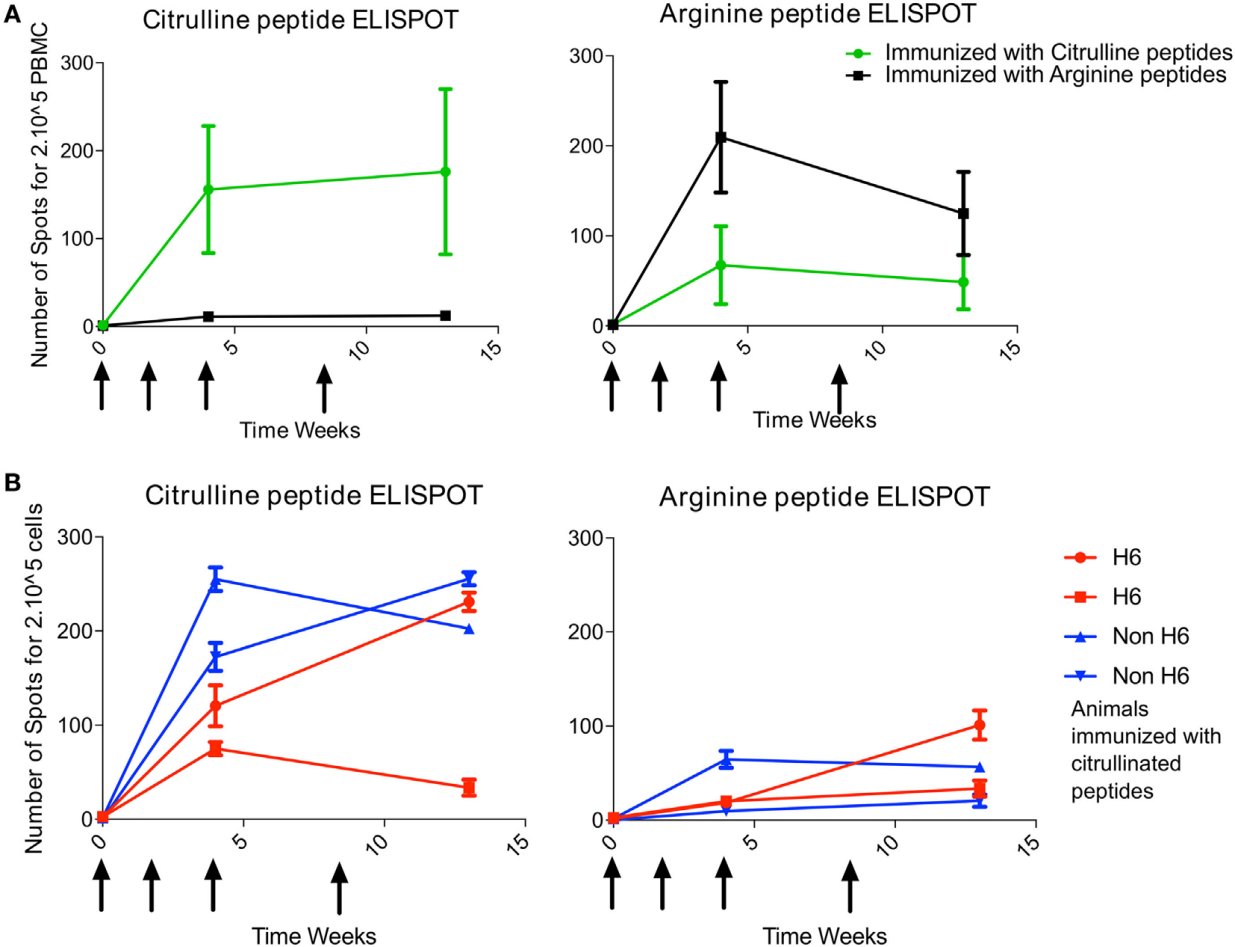
T-cell response to immunization is citrulline dependent. Animals were immunized at weeks 0, 2, 4, and 8 (arrows) with pooled citrulline (*n* = 4) or arginine (*n* = 2) peptides: Vim59 and 66, Fg79, and Agg89. **(A)** Antigen-specific T-cell response of PBMCs was assessed by interferon-gamma (IFNγ) ELISPOT directed against the pool of the same citrullinated or arginine peptides. Data are mean ± SD values for individual animals (performed in triplicate). **(B)** H6 (*n* = 2) and non-H6 (*n* = 2) macaques were immunized with pooled Vim59 and 66, Fg79, and Agg89 peptides at weeks 0, 2, 4, and 8 (arrows). Antigen-specific T-cell response of PBMCs was assessed by IFNγ ELISPOT directed against the pool of the same citrullinated or arginine peptides. Data are mean ± SD for individual animals (performed in triplicate). PBMC, peripheral blood mononuclear cell; ELISPOT, Enzyme Linked Immunospot.

At 4 and 13 weeks’ postimmunization, T-cell response to the pool of citrullinated peptides was greater for animals immunized with the citrullinated peptides (4/4 animals) than arginine peptides (0/2 animals) (Figure 1A). Likewise, T-cell response to the pool of arginine peptides was greater for animals immunized with the arginine version of the peptides (2/2 animals) than the citrulline version (1/4 animals) (Figure 1A). Therefore, after intradermal immunization, the T-cell response was rather citrulline- or arginine-specific depending on the type of peptides used for immunization. Analysis of responses to individual peptides showed a high response to citrullinated Vim59 and native Agg89, whereas all animals showed low or no response to citrullinated or native Fg79-91 (data not shown). Of note, the magnitude of the T-cell response was similar for H6 and non-H6 carriers (Figure 1B). Thus, H6 and non-H6 animals immunized with citrullinated peptides were pooled in the following experiments.

### B-Cell Response to Citrullinated Peptides Is Largely Cross-reactive

**Figure 2:**
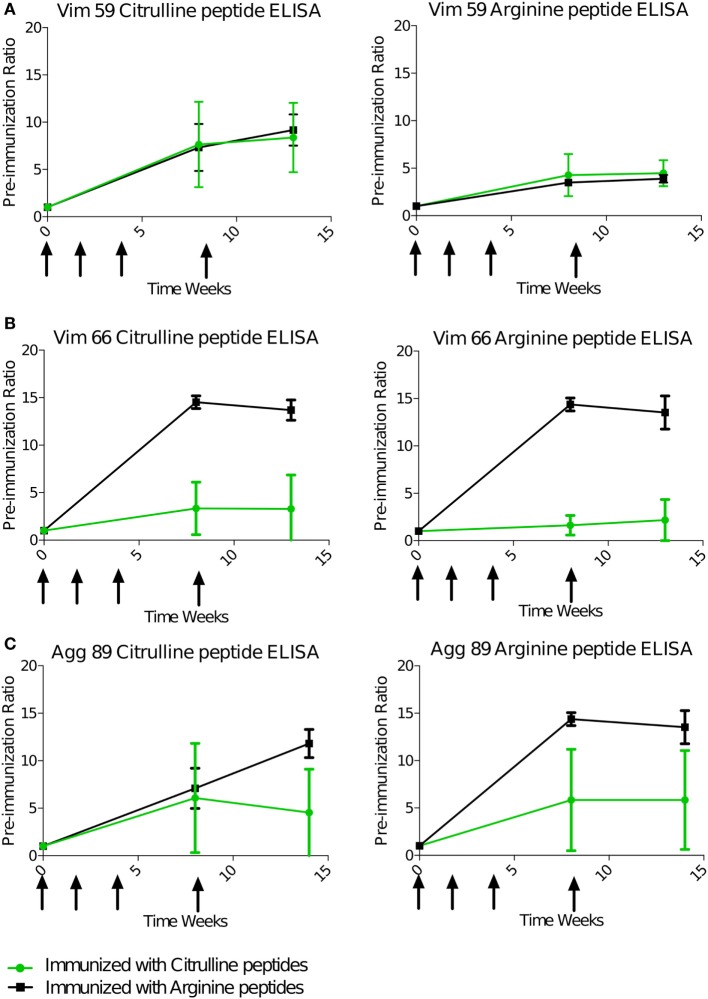
B-cell response is cross-reactive to citrulline and arginine. Animals were immunized with pooled citrulline (*n* = 4) or arginine (*n* = 2), Vim59 and 66, Fg79, and Agg89 peptides at weeks 0, 2, 4, and 8 (arrows). Antibody levels in the serum were assessed by ELISA directed against Vim59 **(A)**, Vim66 **(B)**, and Agg89 **(C)** with cit (left) or arginine (right) except for Fg79 (no response). Data are mean ± SD ratio to the preimmunization state. cit, citrullinated.

Serum antibody response against the four immunizing peptides was monitored individually during and after the immunization phase using ELISA. As for the T-cell response, the B-cell response, animals immunized with the citrulline or native version of the peptides were compared. All animals displayed a strong IgG response as compared with the preimmunization state (Figure 2). This response appeared between 4 and 6 weeks after the first immunization. For Vim59, the antibody response was systematically stronger against the citrullinated peptide than its arginine version (Figure 2A). However, the response was not exclusively dependent on citrullination because animals immunized with arginine peptides also showed a response against the citrullinated form (Figures 2A–C). In general, and contrary to the T-cell response, the B-cell response showed a high degree of cross-reactivity between antibodies against the citrullinated and the native peptide form, whatever the peptide used for immunization. Cross-reactivity was confirmed using an ELISA competition assay—preincubation with arginine or citrulline peptides inhibited anti-citrullinated response in a similar fashion (Figure S7 in Supplementary Material). Of note, all animals had a low or no response to citrullinated or native Fg79. Similar to the T-cell response, the presence of the shared epitope did not affect the B-cell response against the citrullinated or native version of peptides overall (Figure S8 in Supplementary Material).

### IA Boost of Citrullinated Peptides Induces Mono-Arthritis in Animals Immunized against Citrullinated Peptides

**Figure 3:**
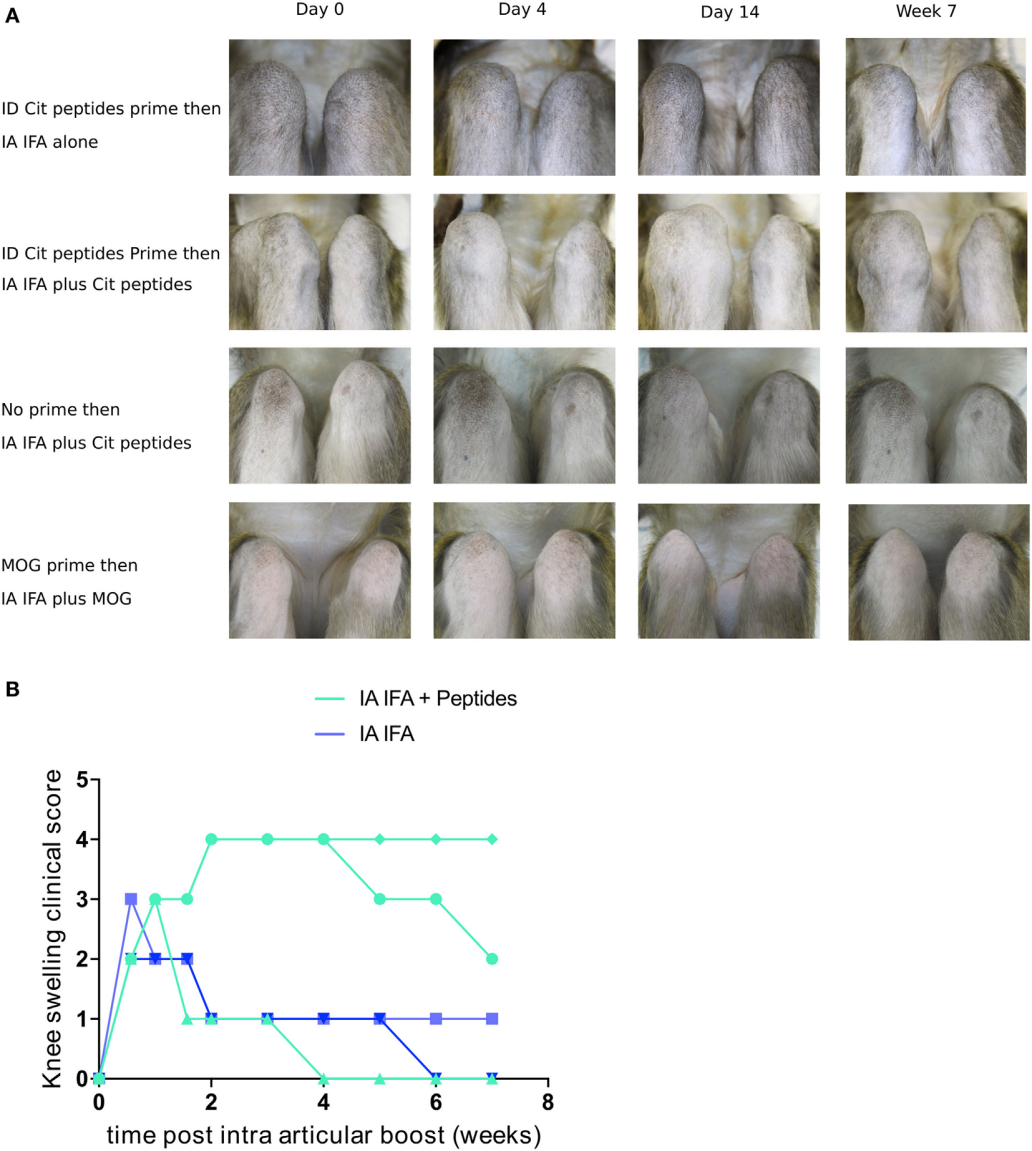
IA boost with citrullinated peptides leads to prolonged articular swelling. **(A)** Animals previously immunized with citrullinated peptides were boosted with IFA alone or citrullinated peptides + IFA in the right knee (upper two panels). Other control groups were (1) naïve animals injected with citrullinated peptides + IFA and (2) rhMOG-primed animals boosted intra-articularly with rhMOG + IFA (lower two panels). Weekly clinical monitoring of both knees is summarized with key time points. Photographs show 90° flexed right knees on the left side of the pictures. **(B)** Semi-quantitative longitudinal clinical evaluation of knee swelling for each animal with previous prime. Comparison between IFA alone and IA with citrullinated peptides + IFA. IFA, incomplete Freund’s adjuvant; IA, intra-articular; cit, citrullinated; rhMOG, recombinant human myelin oligodendrocyte glycoprotein.

During this first systemic immunization phase, despite a significant T- and B-cell response, no clinical manifestations of arthritis were noted. Because in humans, ACPA positivity can be asymptomatic for years before the occurrence of arthritis, second hits may be required to induce arthritis in immunized humans or animals. As a second hit, we chose unilateral injection of citrullinated peptides in the right knee 30 weeks after initial immunization. Five of the six animals previously immunized with citrullinated (*n* = 3) or arginine (*n* = 2) peptides were available for this second step. IA injection of citrullinated peptides without IFA did not induce clinical signs or local immune response. Then, previously immunized animals were unilaterally injected in the right knee with IFA alone (*n* = 2) or citrullinated peptides + IFA (*n* = 3). IFA alone led to a 4-day unilateral knee swelling. Conversely, with IA injection of citrullinated peptides + IFA, two of three animals showed a 7-week unilateral swelling with joint effusion (Figures 3A,B). No other joint was clinically involved. This prolonged mono-arthritis resulted from both previous systemic immunization with citrullinated peptides and knee injection of citrullinated peptides + IFA. Indeed, the same knee injection of citrullinated peptides in two non-immunized animals led to a 4-day transient swelling similar to IFA injection alone.

To study the peptide specificity, IA injection of rhMOG + IFA in two animals previously immunized with rhMOG induced only mild transient swelling for 4 days, similar to that with IFA alone (Figure 3A). Finally, IA immunization with arginine peptides + IFA in a systemically citrullinated primed animal did not yield prolonged swelling. Therefore, only specific and citrullinated antigens could induce prolonged arthritis in an animal immunized against this antigen.

Among the three animals with an IA boost with citrullinated peptides, the two showing prolonged mono-arthritis were H6-positive and the one showing only a short episode of inflammation was not.

### IA Boost with Citrullinated Peptides Induces a High Systemic Immune Response

**Figure 4:**
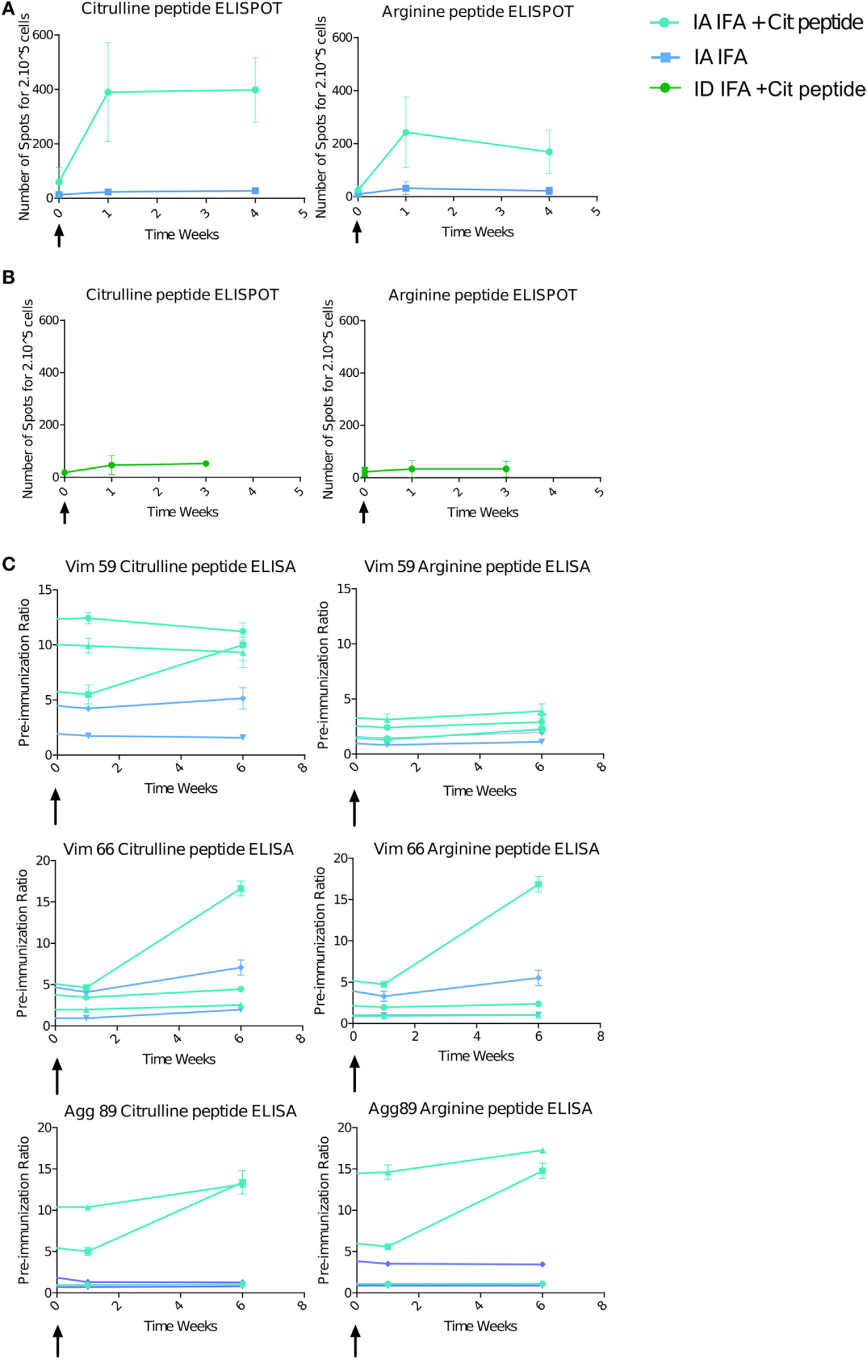
Intra-articular (IA) boost with citrullinated peptides and incomplete Freund’s adjuvant (IFA) induces a strong immune response. At 30 weeks’ post-initial prime, animals received an IA boost with pooled citrullinated Vim59 and 66, Fg79, and Agg89 plus IFA (*n* = 3) or IFA alone (*n* = 2). **(A)** T-cell response assessed by interferon-gamma (IFNγ) Enzyme Linked Immunospot (ELISPOT) against the pooled citrullinated or arginine pool of peptide. Data are mean ± SD values for individual animals (performed in triplicate). **(B)** At 34 weeks’ postimmunization, two animals were intradermally boosted with the same citrullinated peptide pool, and response was assessed by IFNγ ELISPOT. **(C)** ELISA of B-cell response against Vim59 and 66, Fg79 and Agg89 in their citrullinated and arginine form. Data are mean ± SD values for individual animals (performed in triplicate). The time scale is shown as time post-IA injection.

The T-cell response was striking with IA boost with citrullinated peptides + IFA versus IFA alone (3/3 vs 0/2 animals) (Figure 4A). The magnitude of the response after IA boost was two to six times higher than after the intradermal prime, and three boosts with similar quantities of peptides and after late intradermal boost (Figure 4B). This response retained a degree of specificity to citrullinated peptides, with a higher response against these citrullinated peptides versus the pool of arginine peptides. Control animals receiving IA IFA alone showed no response. The B-cell response, which was high before the IA boost, was increased in one of the three animals receiving an IA boost with citrullinated peptides + IFA and in none of the others (Figure 4C).

The striking immune activation after the IA boost with citrullinated peptides + IFA but not IFA alone suggests that the prolonged articular swelling was due to an immune response against the citrullinated peptides, whereas the transient swelling was due to a non-specific inflammatory response to IFA.

**Figure 5:**
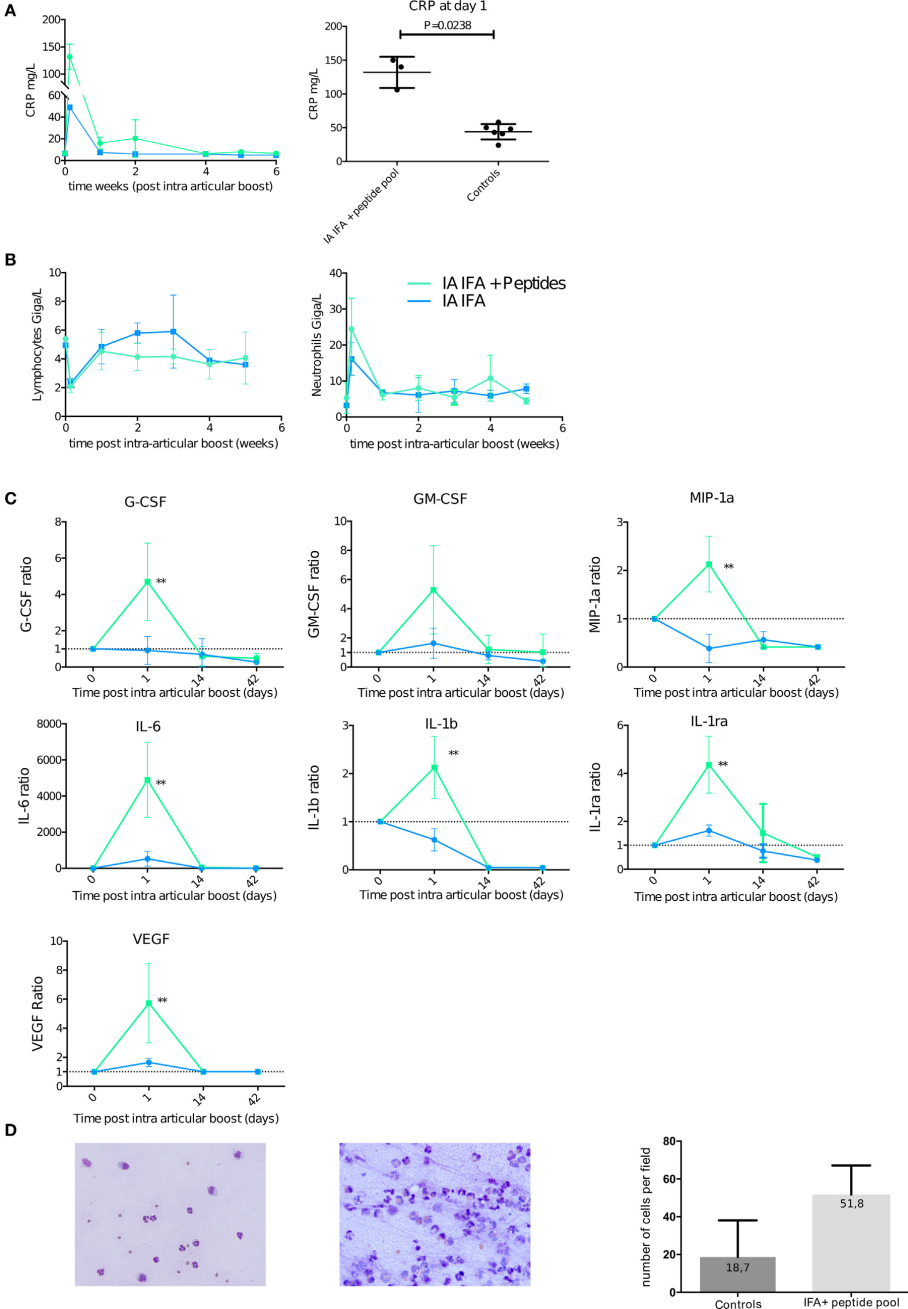
Intra-articular (IA) boost leads to systemic inflammation favoring neutrophilic joint effusion. At 30 weeks post-initial prime, animals received an IA boost with pooled citrullinated Vim59 and 66, Fg79, and Agg89 plus incomplete Freund’s adjuvant (IFA) (*n* = 3) or IFA alone (*n* = 2) as a control. **(A)** Left panel: Plasma C-reactive protein (CRP) level with citrullinated peptides + IFA and IFA alone. Right panel: Plasma CRP level for citrullinated peptides + IFA and all controls at day 1 post-IA boost (Mann–Whitney test). **(B)** Complete blood counts of lymphocyte and neutrophils after IA boost with citrullinated peptides + IFA and IFA alone. **(C)** Non-human primate multiplex 23-cytokine array after IA boost with citrullinated peptides + IFA and IFA alone. Data are ratio of levels to the pre-IA injection level. **P < 0.001 Mann–Whitney test. **(D)** Synovial fluid smears stained with May-Grünwald Giemsa showing typical cellular counts of transient swelling with IFA alone 4 days post-IA injection (left panel) and citrullinated peptides + IFA 14 days post-IA boost (middle panel). 20× magnification. Quantification of at least five high power fields of synovial fluid smears comparing animals with transient swelling on IFA injection (controls) and those who received citrullinated peptides + IFA and presented prolonged swelling. Data are mean ± SD.

This finding is further emphasized by the level of systemic inflammation measured by CRP level in serum at day 1 after the boost. CRP level was higher for animals that received an IA boost with citrullinated peptides + IFA than IFA alone (3/3 animals > 100 mg/mL vs 2/2 animals < 60 mg/mL). rhMOG + IFA or citrullinated peptides + IFA, without a previous prime; all displayed significantly lower levels of CRP (Figure 5A).

This increase in CRP level occurred at the same time (day 1) as a reduced lymphocyte count and an increased neutrophil cell count in blood (Figure 5B). Among a panel of 23 analyzed cytokines (Table S5 in Supplementary Material), a significant increase in ratio of circulating cytokines on day 1 to the pre-IA injection level was limited to proinflammatory and angiogenic cytokines [interleukin (IL)-6, IL-1B, IL1-RA, and VEGF] and granulocyte-stimulating chemokines (GM-CSF and MIP1A) in animals that received an IA boost with citrullinated peptides + IFA versus IFA alone (Figure 5C).

### IA Boost with Citrullinated Peptides Induces Mono-Arthritis

Articular swelling was confirmed by taping effusion liquid. During the first phase of aspecific swelling that appeared in most animals, low cellularity with a predominance of granulocytes occurred (Figure 5D left panel). At later times (7–14 days postinjection), animals with prolonged swelling upon IFA + peptide injection presented high cellularity (Figure 5D middle and right panel).

**Figure 6:**
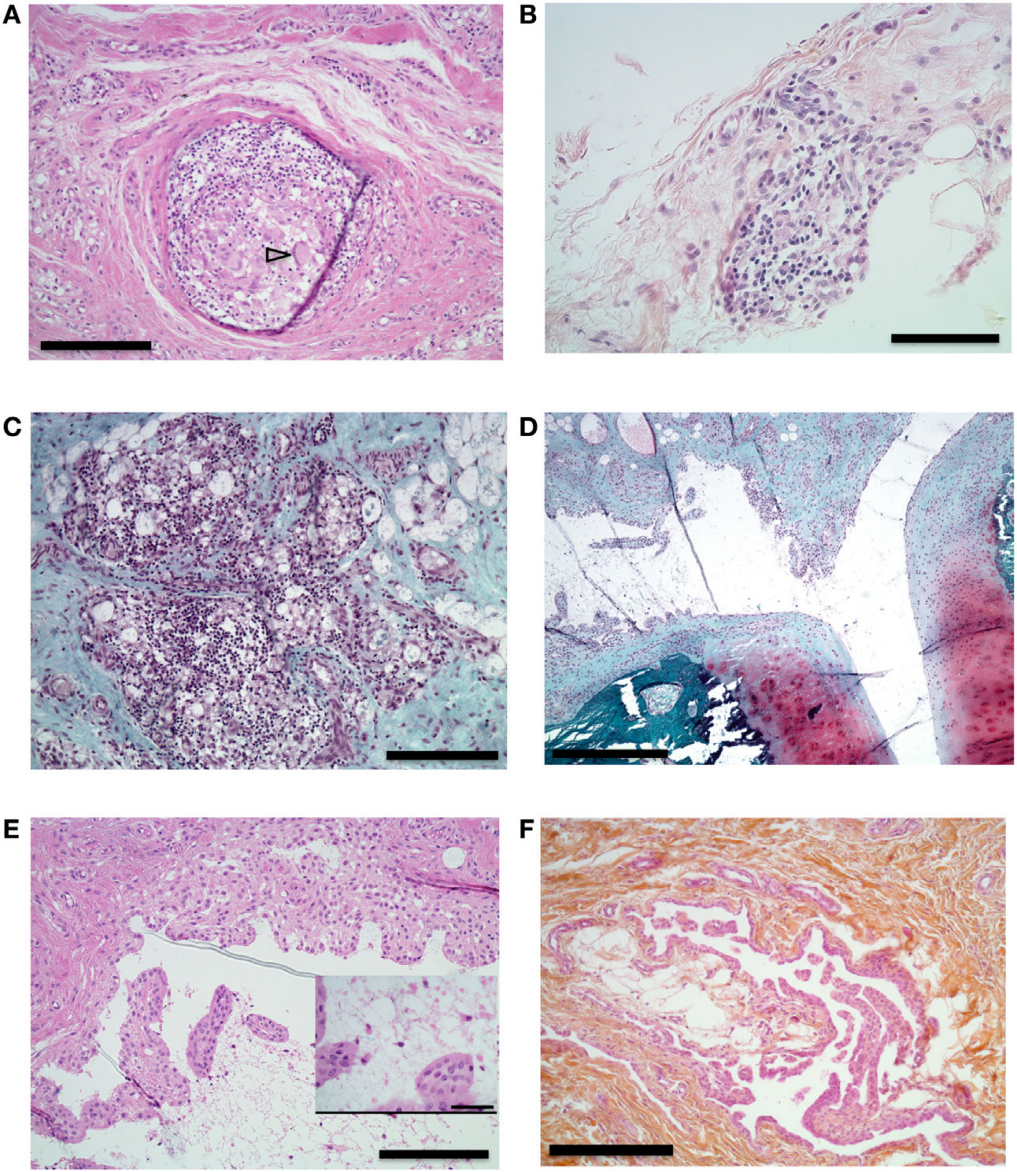
Histological analysis of synovial proliferation. Sections with hematoxylin eosin **(A,B,E,F)** or safranin O and Fast Green **(C,D)** stain. **(A)** Synovial membrane of knee of an animal that received intra-articular (IA) injection containing incomplete Freund’s adjuvant (IFA). Arrowhead shows multinucleated gigantic cell. 10× magnification, bar 200 µm. **(B)** Synovial membrane of right knee of an animal that received IA boost with citrullinated peptides + IFA and presented prolonged swelling. 20× magnification, bar 100 µm. **(C)** Knee synovial membrane. 10× magnification, bar 200 µm. **(D)** Knee synovial membrane, bone, and cartilage of the same animal. 4× magnification, bar 500 µm. **(E)** Right knee synovial membrane of an animal that received an IA boost with citrullinated peptides + IFA and presented prolonged swelling. Insert shows fibrin and leukocyte in the synovial cleft. 10× magnification, bar: 200 µm, insert bar 50 µm. **(F)** Section of metacarpophalangeal joint synovial membrane of an animal without clinically identified manifestation on these joints. 10× magnification bar 200 µm.

Histological analysis of the synovial tissue of the injected knee performed at euthanasia 15 weeks after articular challenge revealed different types of lesions in the injected knee without any clear differences between an IA boost with citrullinated peptides + IFA or IFA alone: unspecific adjuvant-related granulomas (Figure 6A), diffuse mononuclear-cell and granulocyte infiltration of the synovial layer (Figure 6B) with lymphocyte infiltration foci (Figure 6C), and synovial proliferation (Figures 6D,E) without any bone or cartilage lesions (Figure 6D). Finally, systematic sampling of metacarpophalangeal and metatarsophalangeal joints revealed synovial proliferation in two animals with citrullinated peptides + IFA injection (Figure 6F): the animal that died prematurely and thus did not receive the IA boost and an animal with prolonged knee swelling after IA boost with citrullinated peptides + IFA. These last histological lesions were present despite the absence of clinical swelling in these joints.

## Discussion

In this study, we show that intradermal immunization followed by an IA boost with citrullinated peptides can induce a prolonged local synovitis in macaques. This persistent mono-arthritis was restricted to animals previously primed intradermally with a pool of four citrullinated peptides (Vim59-71, Vim66-78, Fg79-91, and Agg89-103). Our model displays many aspects of RA including clinical swelling and pertinent biological modifications with the limitation of a mono-articular disease. Synovial effusion parallels the human disease as it shows high neutrophil predominance.

This study shows clear evidence that the T-cell response against injected peptides is specific to citrullinated peptides. By contrast, the B-cell response to the peptides was largely cross-reactive between citrullinated and native peptides. Citrullinated peptides that react with specific T cells in RA patients may not be able to induce a citrulline-specific B-cell response in macaques. Of note, these peptides present only one posttranslational modification from arginine to citrulline. The cross-reactivity of the B-cell response could simply result from reactivity to epitopes independent of arginine/citrulline and shared by each form of the peptides. However, even in human disease, cross-reactivity of the B-cell response between citrullinated and arginine peptides has been frequently described. In the natural history of the disease, antibodies to some citrullinated peptides can be preceded by antibodies against the arginine counterpart (14, 15). This is also true in mice, because immunization with alpha-enolase induces arthritis with the citrullinated or native forms of the protein (16). Our model forces the generation of antibodies to citrullinated peptides as compared with the natural history of the disease and might need other stimuli to select the clones directed to citrullinated epitopes. Interestingly, in human RA, the clinical signs could appear only after maturation of the B-cell response by epitope spreading against different targets of citrullinated peptides resulting from somatic mutations (17). This maturation might be accompanied by the disappearance of the response to native peptides. The absence of maturation of ACPAs in our model could explain the absence of clinical signs in the first phase of the study before the IA boost.

We based our model on the identity of the H6 genotype of the macaques with one of the human shared epitopes—HLA DRB 01*01 for the 70–74 amino acids (QRRAA). However, comparison of the B- and T-cell response to the peptides did not show a clear effect of the H6 haplotype on the magnitude of the immune response to citrullinated peptides measured as specific antibody titers or specific IFNγ ELISPOT response, respectively. This could be considered surprising given what is usually admitted that there is a better presentation of citrullinated peptides by the shared epitope However, this assumption based on only two studies (3, 5) is still debated. Another study (18) that systematically analyzed T-cell reactivity to peptides derived from the whole alpha and beta chains of fibrinogen in RA patients did not confirm these results and showed that depending on the peptide, the citrullinated form can be more, equally or less reactive than its arginine counterpart. Finally, since we did not use anti-MHC blocking antibodies to block the reaction, we cannot eliminate the possibility that the IFN production we found could have been secreted by T cells not activated through MHC or by other PBMC like NK cells.

Moreover, a recent study of twins showed that bearing the shared epitope most likely favors declaring clinical signs of ACPA-positive RA [odds ratio (OR) 5.65 (95% CI 3.03–10.52)] rather than developing asymptomatic ACPAs alone [OR 1.85 (1.41–2.42)] (19). Among the three animals in our study with an IA boost of citrullinated peptides, only the two H6-positive animals showed prolonged articular swelling. Another explanation for the absence of the H6-carriage effect is that recent literature has highlighted the importance of the presence of the valine in the 11th position (20) with a relative influence of the 70–74 amino acids. The H6 haplotype displays a phenylalanine rather than a valine in the 11th position. Moreover, heterozygous carriage of the shared epitope (as for our animals) only moderately increases the risk of ACPAs [OR 1.85 (1.42–2.42)] as compared with homozygous carriers [OR 3.74 (2.7–5.18)] (19). Finally, interpretations about the effect of the H6 carriage must remain cautious because we only studied two H6 and two non-H6 animals injected with citrullinated peptides. A larger number of animals in each group would be needed to confirm these results, but studies on macaque are not easy to conduct.

One of the most interesting results of our study is the requirement of a multistep process to induce clinical symptoms, in favor of the second-hit hypothesis (21). The IA injection of the citrullinated peptides that had been used for the intradermic prime was chosen as a second hit. Surprisingly, the T-cell response induced by the IA boost was markedly more efficient than an intradermal boost. A similar strong systemic response has been described in staphylococcal septic arthritis (22, 23). However, a simple articular injection of peptides without previous immunization was inefficient to induce clinical signs. The requirement of adjuvant IFA suggests that transient local inflammation is necessary to trigger a prolonged effect linked to a specific immune response. With this IA boost strategy, long-term mono-arthritis developed in two of the three animals injected with citrullinated peptides + IFA. This clinical manifestation displayed many features of the human disease, including swelling and articular effusion, but limited to a single joint. Even if IFA was required, the role of a specific response against anti-citrullinated peptides was demonstrated by (1) an extended duration of arthritis as compared with the transient inflammation induced by IFA alone, (2) the requirement for a previous intradermic immunization with citrullinated peptides, and (3) the absence of prolonged arthritis in animals previously intradermally immunized with rhMOG protein and receiving an IA boost with rhMOG + IFA. These findings suggest that the citrullinated proteins may be a tissue-specific articular target on the RA joint inflammation and need to be locally present to perpetuate the clinical response. Indeed, fibrinogen (24), vimentin (25), and aggrecan (10) but not rhMOG are present in the joint and are citrullinated in the presence of inflammation, which perpetuates the immune response and possibly the clinical manifestation. Antigen specificity and not non-specific Arthus phenomenon or immune complexes seem involved here because the same course of immunization with rhMOG did not induce arthritis.

The main limitation of the current model is its restriction to clinical mono-arthritis despite a specific T-cell response associated with a strong systemic immune response after IA injection. However, histological analysis showed synovial tissue proliferation in joints distant from the knee, which suggests initiation of diffuse joint inflammation similar to human RA. The systemic inflammation, assessed by increased blood neutrophil count and serum CRP level, does not seem sufficient to cause clinically relevant joint inflammation in other non-injected joints. Neutrophils, which are probably implicated in the pathophysiology of RA (26), were present in the affected joint of our model. Such an acute inflammation leading to neutrophil extracellular trap (NET) or NETosis, could enhance citrullination of local proteins, making them targets for ACPAs and permitting the formation of ACPA immune complexes, responsible for the perpetuation of local inflammation (27, 28). To improve this model, an objective could be to initiate joint inflammation using viral infection to favor migration of neutrophils to other joints. IL-8 could be a good candidate for this spread because it attracts neutrophils and was found released by patient-derived osteoclasts (29). However, despite the absence of clinical polyarthritis, we observed elevated blood neutrophil count in the two animals with long-term mono-arthritis. As discussed previously, the absence of maturation of the ACPAs could, at least, be part of the explanation. Nevertheless, the histological examination of metacarpophalangeal and metatarsophalangeal joints in two animals immunized with citrullinated peptides displayed a synovial proliferation very similar to what is observed in human RA. This histological abnormality was not clinically detectable. Ultrasonography would thus be required for the follow-up of the animals in further experiments.

## Conclusion

Immunization of macaques with citrullinated peptides followed by an IA boost with the same citrullinated peptides plus IFA induced prolonged mono-arthritis. The presence of the shared epitope did not restrict the T-cell response against citrullinated peptides but seemed to favor the prolonged swelling after the IA boost. This two-step model of prolonged mono-arthritis mimics what could happen in human RA except that the animals develop mono-arthritis and not polyarthritis. Further use of systemic viral infection and a neutrophil chemoattractant might lead to a polyarticular disease. Nevertheless, this model appears interesting to study the events occurring during the preclinical phase of RA from immunization against citrullinated peptides to development of the clinical disease.

## Ethics Statement

This study was carried out in accordance with the recommendations of “European Union Directive 2010/63/EU, 2010.” The protocol was approved by the “Comité Régional d’Ethique sur l’Expérimentation Animale Île de France Sud, Fontenay-Aux-Roses, France.”

## Author Contributions

SB, PR, TL, GN, CS, PC, GS, RG, and XM made substantial contributions to study conception and design, and analysis and interpretation of data and gave final approval of the article. SB, PR, TL, PC, and CS made substantial contributions to acquisition of data. SB, PR, CS, GS, RG, and XM drafted the article for important intellectual content.

## Conflict of Interest Statement

The authors declare that the research was conducted in the absence of any commercial or financial relationships that could be construed as a potential conflict of interest.
