# Supplementary material for: Both Systemic and Intra-articular Immunization with Citrullinated Peptides Are Needed to Induce Arthritis in the Macaque
Source: Front Immunol. 2017 Dec 20;8:1816. doi: 10.3389/fimmu.2017.01816 (PMC5742322; doi:10.3389/fimmu.2017.01816)
Supplement: Supplementary file 1 [file Data_Sheet_1.DOCX]

Supplementary Material

**Both systemic and intra-articular immunization with citrullinated peptides are needed to induce arthritis in the macaque**

Samuel Bitoun ^1*^, Pierre Roques ^2^, Thibaut Larcher^3^, Gaétane Nocturne^1^, Che Serguera^4^, Pascale Chrétien^5^, Guy Serre^6^, Roger Le Grand^2^, Xavier Mariette^1^

*** Correspondence:** Samuel Bitoun: [samuelbitoun@yahoo.fr](mailto:samuelbitoun@yahoo.fr)

**Table S1: Detailed immunization protocols for each animal**

| Animal ID | 1 | 2 | 3 | 4 | 5 | 6 | 7 | 8 | 9 | 10 |
| --- | --- | --- | --- | --- | --- | --- | --- | --- | --- | --- |
| H6 status | **+** | **+** | **+** | **+** | **-** | **-** | **-** | **-** | **-** | **-** |
| Systemic immunization protocol | Cit pep+ Montanide | Cit pep+ Montanide | Arg pep+ Montanide | Arg pep+ Montanide | Cit pep+ Montanide | Cit pep+ Montanide | rhMOG +IFA | rhMOG +IFA | none | none |
| IA injection of cit pep | + | + | + | + | + | NA | no | no | no | no |
| IA immunization protocol | Cit pep +IFA | IFA alone | IFA alone | Cit pep+ IFA | Cit pep+ IFA | NA | rhMOG  +IFA | rhMOG +IFA | Cit pep + IFA | Cit pep + IFA |
| IA immunization protocol 2 in other knee | NA | Arg pep +IFA | Arg pep +IFA | NA | NA | NA | NA | NA | NA | NA |
| observation |  |  |  |  |  | euthanized prematurely |  |  |  |  |

cit pep, citrullinated peptides; IFA, incomplete Freund’s adjuvant; rhMOG, recombinant human myelin oligodendrocyte glycoprotein

**Table S2: Alignment of DRB-1 amino acid shared epitope sequences in human leukocyte antigen (HLA) and cynomolgus macaque antigen (*Mafa*). Cynomolgus haplotype is called H6.**

|  | 70 80 |
| --- | --- |
| HLA-DRB1*01:01 | WNSQKDLLE**QRRAA**VDTYCR |
| HLA-DRB1*04:01 | ---------**-K---**------ |
| MAFA-DRB1*W04:01 | ---------**-----**------ |

**Table S3: Primer and probe sequence required for shared epitope confirmation**

RNA was extracted from leucocytes using an RNA easy kit (Qiagen, France). RT-PCR was performed with forward (F) and reverse (R) primers and the H6 or H2 probe with primers that amplified the sequence from H2 or H6 haplotypes at 56°C for 30 min followed by 5 min at 95°c. PCR involved 15 sec of denaturation at 95°C and 30 sec at 61.2°C for elongation.

| Designation | Nucleotide sequence |
| --- | --- |
| Primer sequence F | CGGTTGCTGGAGAGACACTT |
| Primer sequence R | CACTCACAGAGCAGACCAGG |
| H6 specific probe | AGCAAAAGCGGGCTGCGGTGG |
| H2 Specific probe | AGCAGAAGCGGGGCCAGGTG |

**Table S4:** **Amino acid sequence of the selected peptides in their citrullinated form, with bold R indicating the arginine residue that was converted to citrulline.**

| Protein | Peptide sequence |
| --- | --- |
| Vimentine 59-71 | GVAT**R**SSAVRLR |
| Vimentine 66-78 | SAVRA**R**SSVPGV |
| Fibrinogen alpha 79-91 | QDFTN**R**INKLKNS |
| Aggrecan 89-103 | VATEG**R**VRVNSAYQDK |

**Table S5: Twenty-three cytokines analyzed in the non-human primate multiplex.**

| G-CSF | IL-5 | MCP-1/CCL2 |
| --- | --- | --- |
| GM-CSF | IL-6 | MIP-1a |
| IFNg | IL-8 | MIP-1b |
| IL-2 | IL-12/23 | sCD40L |
| IL-10 | IL-13 | TGFa |
| IL-1b | IL-15 | TNFa |
| IL-1ra | IL-17a | VEGF |
| IL-4 | IL-18 |  |

**Method S6:**

Intra-articular injection procedure was established with animals euthanized for other protocols.

Injection involved an anterior route on a 90° flexed-knee patella with blue color dye. The intra-articular location of the injection was confirmed by dissection.

**Figure S7:** **Study of antibody cross-reactivity by ELISA:**

Serum (1/50 000 dilution) was pre-incubated with citrullinated Vim59 or arginine Vim59 before citrullinated Vim59 ELISA. The obtained optical densities were compared to serum without peptidic incubation at the same dilution using regular citrullinated Vim59 ELISA.

**Figure S8: B-cell response is similar in H6 and non-H6 animals**

H6 (n=2) and non-H6 (n=2) macaques were immunized with citrulline Vim59 and 66, Fg79 and Agg89 at weeks 0, 2, 4, and 8 (arrows). Antibody levels in serum were assessed by ELISA directed against Vim59 (A) Vim66 (B) and Agg89 (C) in their citrullinated (left) or arginine (right) form except for Fg79 (no response). Responses of individual animals are shown as mean ± SD.
